# Burrowing by translocated boodie (*Bettongia lesueur*) populations alters soils but has limited effects on vegetation

**DOI:** 10.1002/ece3.7218

**Published:** 2021-03-02

**Authors:** Bryony J. Palmer, Leonie E. Valentine, Cheryl A. Lohr, Gergana N. Daskalova, Richard J. Hobbs

**Affiliations:** ^1^ School of Biological Sciences University of Western Australia Crawley WA Australia; ^2^ Biodiversity Conservation Science Department of Biodiversity, Conservation and Attractions Kensington WA Australia; ^3^ School of GeoSciences University of Edinburgh Edinburgh UK

**Keywords:** *Bettongia*, digging mammal, ecosystem engineer, reintroduction, restoration, translocation

## Abstract

Digging and burrowing mammals modify soil resources, creating shelter for other animals and influencing vegetation and soil biota. The use of conservation translocations to reinstate the ecosystem functions of digging and burrowing mammals is becoming more common. However, in an increasingly altered world, the roles of translocated populations, and their importance for other species, may be different. Boodies (*Bettongia lesueur*), a commonly translocated species in Australia, construct extensive warrens, but how their warrens affect soil properties and vegetation communities is unknown. We investigated soil properties, vegetation communities, and novel ecosystem elements (specifically non‐native flora and fauna) on boodie warrens at three translocation sites widely distributed across the species’ former range. We found that soil moisture and most soil nutrients were higher, and soil compaction was lower, on warrens in all sites and habitat types. In contrast, there were few substantial changes to vegetation species richness, cover, composition, or productivity. In one habitat type, the cover of shrubs less than 1 m tall was greater on warrens than control plots. At the two sites where non‐native plants were present, their cover was greater, and they were more commonly found on boodie warrens compared to control plots. Fourteen species of native mammals and reptiles were recorded using the warrens, but, where they occurred, the scat of the non‐native rabbit (*Oryctolagus cuniculus*) was also more abundant on the warrens. Together, our results suggest that translocated boodie populations may be benefiting both native and non‐native flora and fauna. Translocated boodies, through the construction of their warrens, substantially alter the sites where they are released, but this does not always reflect their historic ecosystem roles.

## Graphical abstract

Boodies (*Bettongia lesueur*) construct extensive warrens, but how this affects soil properties and vegetation communities at translocation sites is unknown. Here, we investigated soil, vegetation and novel ecosystem elements on boodie warrens of three translocated populations. We found that warrens consistently altered soil properties but there were few substantial changes to vegetation species richness, cover, composition, or productivity. Both native and non‐native species were present on boodie warrens. Translocated boodies substantially alter their release sites but this may not always reflect their historic ecosystem roles.

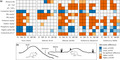

## 1 INTRODUCTION

As ecosystem engineers (sensu Jones et al., 1994), digging mammals influence geomorphological processes (Butler, 1995) and soil resources, creating shelter for other animals (e.g., Dawson et al., 2019; Whittington‐Jones et al., 2011) and affecting vegetation and soil biota (Davidson et al., 2012; Fleming et al., 2014). However, due to complex interactions between the digging species, habitat and soil characteristics, and the extent and longevity of the burrows or warrens, the effects of digging mammals on soil and vegetation properties vary (Mallen‐Cooper et al., 2019). For example, vegetation cover, biomass, and species richness are higher on the burrows and warrens of some species, for example, pocket gophers *Thomomys talpoides* (Grant et al., 1980), pikas *Ochotona pallasi* (Wesche et al., 2007), and Artic ground squirrels *Urocitellus parryii* (Wheeler & Hik, 2013), but lower on those of others such as black‐tailed prairie dogs *Cynomys ludovicianus* (Davidson & Lightfoot, 2006) and Daurian pikas *Ochotona daurica* (Komonen et al., 2003). How the magnitude and direction of the effects of digging mammals on soil resources and vegetation might be altered as environmental conditions change is largely unknown. Burrows could increase in importance as thermal refuges from climate change for other animals (Pike & Mitchell, 2013), and diggings may help rehabilitate degraded areas (Andersen & Macmahon, 1985; Munro et al., 2019). Conversely, digging mammals may accelerate ecosystem change by facilitating the spread or growth of non‐native plants (Larson, 2003; Torres‐Díaz et al., 2011). To effectively manage terrestrial ecosystems, we need to understand what roles digging mammals play in specific locations, and how they interact with novel (i.e., not previously occurring in that ecosystem) elements and conditions.

In an increasingly altered world, translocations are used to improve the conservation status of threatened species and to return species to areas from which they had become extirpated (IUCN/SSC, 2013; Seddon et al., 2014). Translocations are also more frequently being used to restore ecosystems by reinstating ecosystem processes regulated by lost species (Palmer et al., 2020). Ecosystem engineers, such as digging and burrowing mammals, may be particularly useful for this kind of translocation because of their ability to fundamentally restructure ecosystems (Seddon, 2010). However, predicting the effects of translocating an ecosystem engineer will require a thorough understanding of the species’ historical roles in regulating key processes, such as nutrient cycling and plant recruitment, and insight into how these may be altered by the presence of novel ecosystem elements. Since the majority of terrestrial habitats have been, or will be, altered by human activity (Ellis et al., 2010), understanding interactions between ecosystem engineers and novel ecosystem elements is particularly relevant within a translocation context.

Boodies (*Bettongia lesueur*) are medium‐sized (~1 kg), Australian marsupials that forage for underground food resources, creating numerous, shallow digs in the process (Burbidge et al., 2008; Newell, 2008). Uniquely among macropods, boodies also construct complex warren systems (Sander et al., 1997). Boodies once occurred across most of Australia's semi‐arid and arid zones but natural populations are now restricted to three Western Australian islands (Burbidge et al., 2014). Predation by the introduced red fox (*Vulpes vulpes*) and feral cat (*Felis catus*), human persecution and habitat degradation are major factors in their decline, which occurred following European occupation of Australia, with the last boodies recorded on the mainland in the 1960s (Burbidge et al., 2014). To redress their decline, boodies have been translocated to a number of additional islands and fenced, mainland reserves. Conservation concerns continue to drive translocation actions for boodies, but most future translocations are also aiming to restore the species’ historic ecosystem functions (Palmer et al., 2020). However, our understanding of their roles within ecosystems is largely based on the effects of their foraging diggings. Information on the impacts of their warrens on soils and vegetation is limited to a pilot study (Chapman, 2015) and an investigation of long‐abandoned “relict” warrens (Noble et al., 2007). Boodie foraging diggings typically cover less than half a square meter and may persist for several months or, sometimes, years (B. Palmer *pers. obs*.) while boodie warrens can be more than 90 m in diameter and can persist for decades after abandonment (Burbidge et al., 2007). Because diggings and warrens occur over different temporal and spatial scales, more detailed information about the impacts of their warrens is required to complete our knowledge of boodies’ ecosystem functions.

To inform future translocations and to clarify if, and how, translocated boodies direct ecosystem change, we need to quantify how boodie warrens affect ecosystems, and how they interact with novel ecosystem elements. In this study we address four primary questions designed to elucidate the importance of boodie warrens in ecosystems: (a) How does the construction of boodie warrens alter soil properties and ground cover?, (b) How does the construction of boodie warrens alter vegetation communities and plant productivity?, (c) How do novel ecosystem elements, specifically non‐native plants and animals, interact with boodie warrens?, and (d) How do the structural characteristics of boodie warrens vary with habitat, and how does this influence their effects on soils and vegetation communities? To answer these questions, we identified boodie warrens at three sites, and measured soil properties, ground cover, and vegetation species richness, cover, composition and productivity. We also recorded evidence of other vertebrates using the warrens, and quantified warren density, size and activity levels.

## 2 MATERIALS AND METHODS

### 2.1 Study sites

**FIGURE 1:**
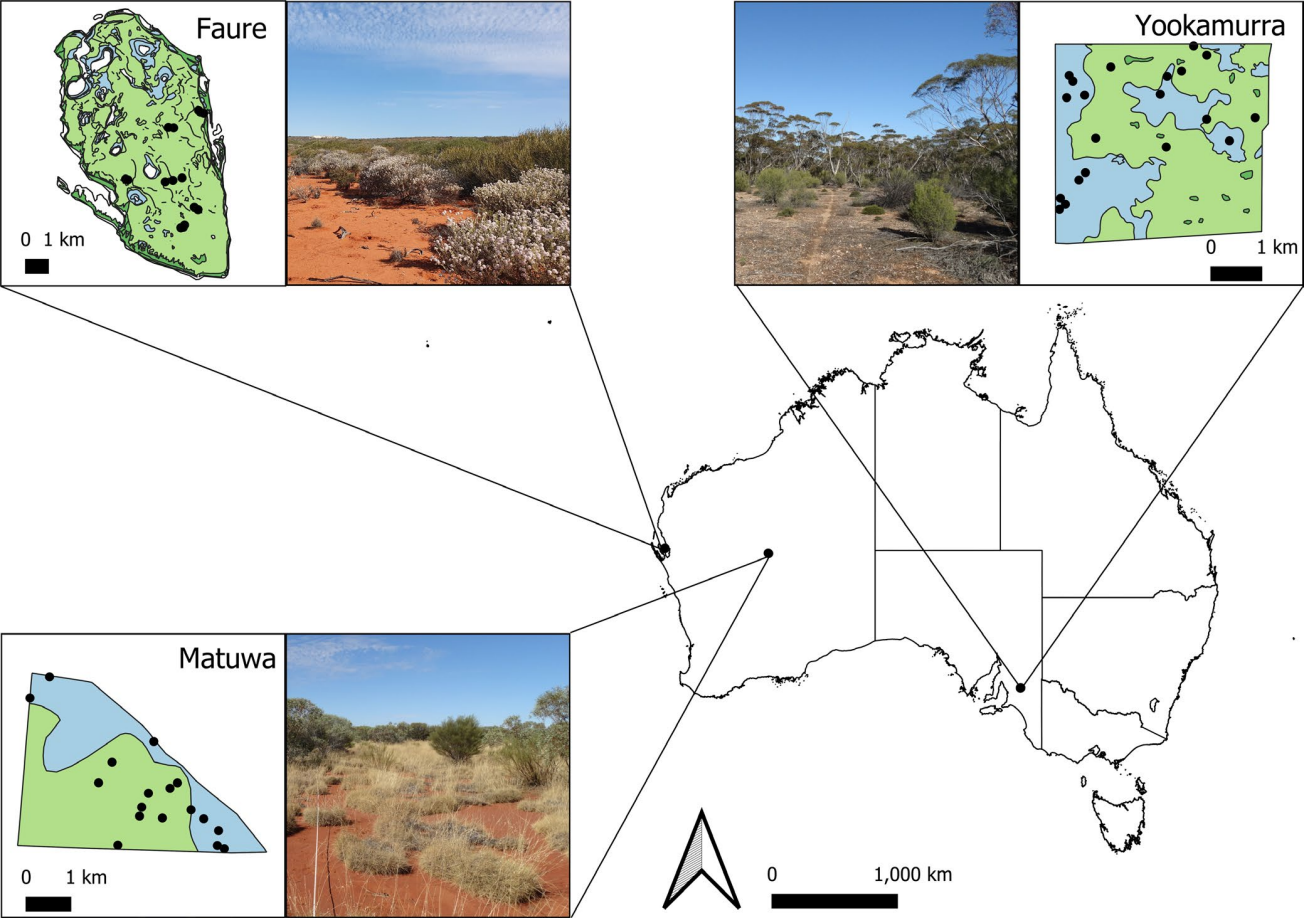
Our study included three boodie translocation sites spanning a range of environmental conditions and translocation histories. Inset maps show the major habitat types (Faure: green—acacia, blue—all other habitats; Yookamurra: green—mallee, blue—myoporum; Matuwa: green—spinifex, blue—shrub) and location of the studied boodie warrens (black circles) at each site. Inset photographs (credit: B Palmer) show examples of the acacia habitat at Faure, the myoporum habitat at Yookamurra, and the spinifex habitat at Matuwa. Only the fenced enclosures at Matuwa and Yookamurra are shown

Research was conducted at three sites, one in eastern South Australia and two in Western Australia, supporting translocated boodie populations: Yookamurra Wildlife Sanctuary (hereafter “Yookamurra”), Matuwa‐Kurarra Kurarra Indigenous Protected Area (“Matuwa”) and Faure Island Wildlife Sanctuary (“Faure”; Figure 1). The sites are all former pastoral leases currently managed for conservation and/or cultural purposes but have different climates, habitat types and boodie translocation histories (Table 1). The boodie populations at both Matuwa and Yookamurra inhabit 1,100 ha fenced reserves from which all mammalian predators have been removed; Faure is a 4,500 ha island which is also free from mammalian predators. Boodies formerly inhabited Matuwa and Yookamurra, and at Matuwa relict boodie warrens can still be observed throughout areas with calcareous soils. There is no evidence for the former presence of boodies on Faure, but they were present on the adjacent mainland at the time of European occupation.

**TABLE 1:** Study site characteristics

		Yookamurra	Matuwa	Faure Island
Sanctuary attributes	Location	−34.519, 139.458	−26.199, 121.360	−25.853, 113.891
	Property size	5,108 ha	244 000 ha	4,561 ha
	Year conservation management commenced	1989	2000	2000
Climate	Mean annual rainfall	270 mm	262 mm	208 mm
	Mean winter minimum	4.7°C	5.4°C	10.5°C
	Mean summer maximum	30.2°C	38°C	34.8°C
Vegetation and soils	Vegetation structure	Mallee woodlands, Myoporum shrublands	Spinifex grassland, Acacia shrubland	Acacia shrubland
	Main overstory species	Eucalyptus	Eucalyptus, Acacia	Acacia
	Soil type	Calcarosol	Calcarosol/tenosol	Tenesol
Boodie attributes	Area inhabited	1,100 ha	1,100 ha	4,561 ha
	Year of first successful release	2007	2010	2001
	Population density (2018–9)	0.21 ha^−1^	0.27 ha^−1^	3.3 ha^−1^
Other burrowing species present		*Macrotis lagotis, Varanus spp*.	*Macrotis lagotis, Varanus* spp.*, Oryctolagus cuniculus*	*Varanus gouldii*

Note

Data sources: Sanctuary attributes, vegetation, boodie attributes, other burrowing species—Australian Wildlife Conservancy (Yookamurra and Faure) and the Western Australian Department of Biodiversity, Conservation and Attractions (Matuwa) unpublished data; Climate—Bureau of Meteorology (2019), except Yookamurra rainfall—AWC unpublished data; Soil type—Ashton and McKenzie (2001).

### 2.2 Warren identification

We walked transects to locate warrens constructed by the translocated boodie populations in August 2018 (Yookamurra), and in March (Matuwa) and September (Faure) 2019. An even number of transects, running at right angles from vehicle access tracks, were randomly located in the dominant vegetation types at each property: mallee woodlands on sandy soils (“mallee”) and myoporum shrublands on calcareous soils (“myoporum”) at Yookamurra, spinifex grasslands on red sands (“spinifex”) and acacia shrublands on calcareous soils (“shrub”) at Matuwa, and acacia shrublands on red sands (“acacia”) at Faure. In total, approximately 20%, 30% and 1% of the areas inhabited by boodies were searched at Yookamurra, Matuwa and Faure respectively. Boodie warrens found along the transects were differentiated from the burrows (i.e., single entrance holes) or warrens (i.e., networks of interconnected burrows) of other co‐occurring burrowing species using a combination of warren/burrow morphology and animal tracks and scats. Boodie warren locations were marked on a GPS.

We measured the diameter of each identified boodie warren along, and at right angles to, its longest axis. We also counted the number of active and inactive entrances on each warren. Active entrances showed evidence of recent use including freshly disturbed soil, tracks or fresh scats. Inactive entrances had no fresh animal sign, had debris blocking the entrance, or were partially or completely collapsed. Warrens selected for further study had at least three active burrow entrances, were undisturbed by human interference, were greater than 200 m away from another selected warren and were evenly distributed among the dominant vegetation types at each site. This resulted in slightly different sample sizes for each site: Faure *n* = 15, Yookamurra *n* = 20, Matuwa *n* = 17. We established a paired control plot, of the same dimensions as the warren, 50–100 m from each warren in the same habitat type.

### 2.3 Soil assessments

**FIGURE 2:**
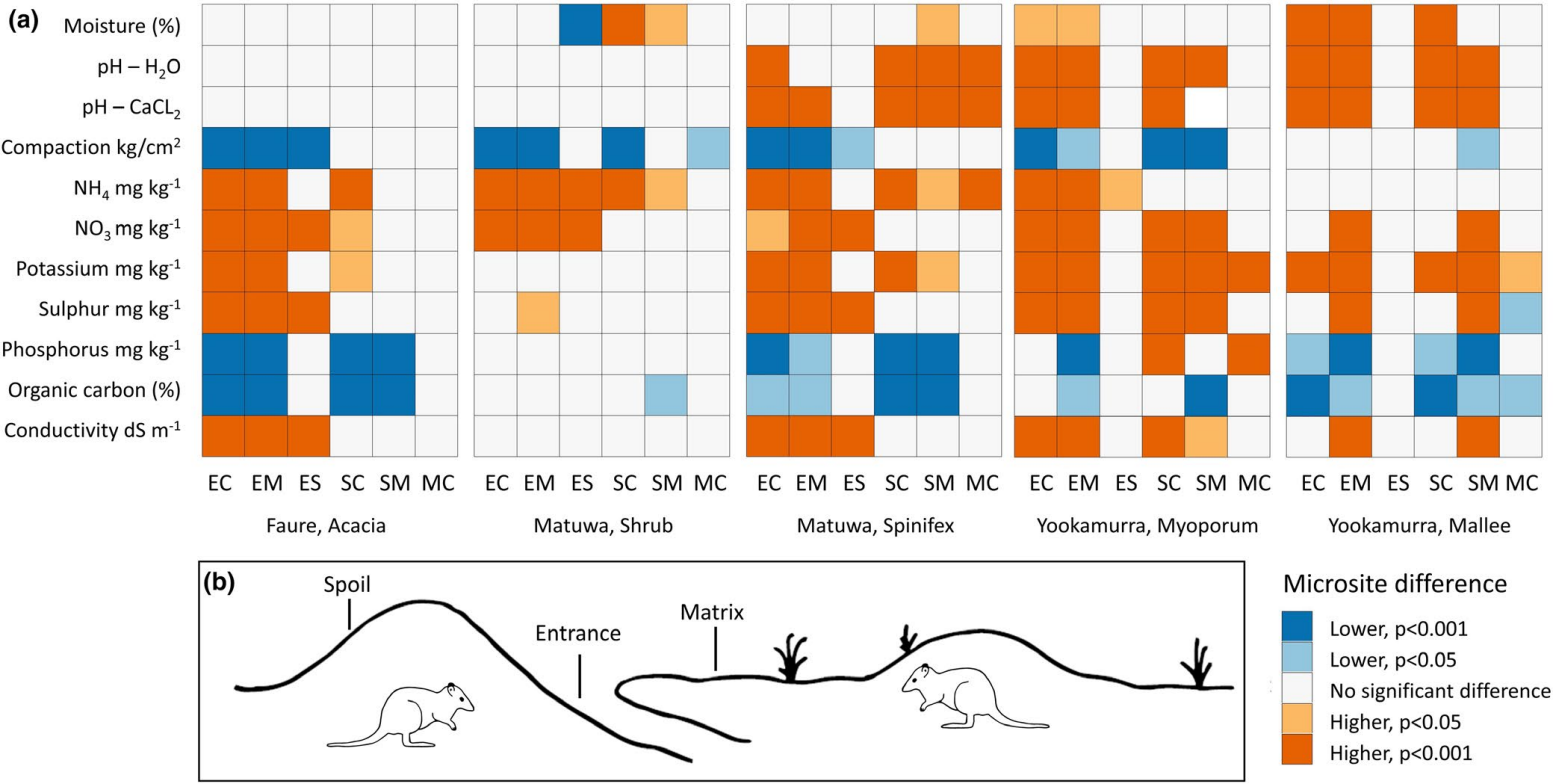
(a) Boodie warrens modify soil properties in largely consistent ways across three sites and five habitat types. Plot shows the results (*p* values) of generalized linear mixed‐effects models comparing soil properties at microsites on boodie warrens (entrances, spoils, matrix) and at paired undisturbed controls. Blue cells indicate the value for first microsite in the pair was significantly lower than the second microsite. Orange cells indicate the value for the first microsite in the pair was significantly higher than the second microsite. The microsite pairs are denoted by the codes: EC—entrance and control, EM—entrance and matrix, ES—entrance and spoil, SC—spoil and control, SM—spoil and matrix, MC—matrix and control. (b) A schematic representation of a boodie warren showing the locations of the warren microsites: Entrances are the holes through which the boodies enter and exit the warren, spoils are the mounds of loose soil that accumulate through the excavation of burrows, and the matrix is the relatively undisturbed area of ground interspersed between entrances and spoils within the warren boundaries. Undisturbed control sites were located 50–100 m from each warren

Soil assessments, that is, moisture and compaction measurements and sampling to assess nutrient content, pH, and conductivity, were conducted in August 2018 at Yookamurra, March 2019 at Matuwa, and September 2019 at Faure. On warrens, soils were assessed at three microsites: burrow entrances, spoil piles and the intervening matrix (Figure 2b). Burrow entrances (hereafter “entrances”) are the holes through which the boodies enter and exit the warren. Spoil piles (“spoils”) are the mounds of soil that accumulate through the excavation of burrows. The intervening matrix (“matrix”) is the relatively undisturbed area of ground interspersed between burrow entrances and spoil piles within the warren boundaries. We assessed three replicates for each microsite type at each warren and selected only active entrances and spoils. Three random locations were selected for assessment within each control plot (“controls”) by blindly tossing a small pebble from the center of the plot.

We measured soil moisture (%) to a depth of 10 cm using a Hydrosense II soil moisture reader. We measured soil compaction (kg/cm^2^) using a Geotester pocket penetrometer; we averaged the results of ten compaction readings from each microsite replicate to get the final compaction reading for that replicate, as per the manufacturer's recommendations. We collected a soil sample, from a depth of 10 cm, from each warren microsite and control replicate. Replicates from each microsite or control were then pooled for analysis. The CSBP Soil and Plant Laboratory conducted standard tests for total carbon (%), nitrate nitrogen (mg/kg), ammonium nitrogen (mg/kg), phosphorus (mg/kg), potassium (mg/kg), sulfur (mg/kg), electrical conductivity (dS/m) and pH (CSBP Lab, 2019).

### 2.4 Vegetation and ground cover sampling

Vegetation data were collected at Yookamurra in August 2018, at Matuwa in March 2019 and Faure in September 2019. These sampling periods fall within the usual growing season at each site. All three sites received less than average rainfall in the 2 years prior to the surveys (Bureau of Meteorology, 2019). At each warren, two perpendicular transects were established, with one transect running along the longest axis of the warren. The transects were centered on the warren mid‐point and extended beyond the edge of the warren by three meters for every one meter on the warren to encompass the effects of underground burrows that extended beyond the visible surface disturbance. Transects of the same length and compass orientation were established at each paired control plot.

The same observer assessed vegetation and ground cover in 50 cm^2^ quadrats, placed at intervals along each transect, at all locations. Because warrens varied in size, quadrat spacing was adjusted so that between 10 and 25 quadrats were sampled per warren. A greater number of quadrats were sampled on larger warrens. The number and spacing of quadrats for control plots were matched to their paired warren. The area of ground disturbed by mammals and the cover of bare ground, leaf litter and cryptogamic crust were estimated to the nearest whole percent within each quadrat. Plants that had any portion of their foliage within a quadrat were identified to species or genus level where possible and their percent cover estimated. Species that had a total cover estimate of less than 1% were assigned a value of 0.5% for analysis. Ground and plant cover estimates summed to 100% for each quadrat but area disturbed by mammals was assessed separately (e.g., where leaf litter overlaid patches of cryptogamic crust the area was designated as leaf litter not cryptogamic crust, but where leaf litter overlaid areas of mammal disturbed ground the area was included in both the disturbed ground and leaf litter estimates). Animal scats found within each quadrat were counted and identified to species. While we were conducting the soil and vegetation surveys, we additionally recorded all vertebrate species we observed using a warren, that is, sheltering within, or entering or exiting a burrow.

### 2.5 Productivity estimates

Aerial vegetation surveys were conducted at Yookamurra in March 2019, Faure in September 2019 and at Matuwa in October 2019. Aerial imagery was collected by a DJI Phantom 4 Pro V2.0 drone equipped with a Sentera High Precision NDVI Single sensor, flown at 5 m/s at a height of 50 m. This resulted in images with a resolution of 0.05 m/pixel. Images were collected every two seconds. Paired warren and control plots were included in the same flight. At Matuwa and Faure flight conditions were consistently clear and sunny; at Yookamurra some light clouds were experienced at times. Flights were primarily conducted within 2 hr of solar noon, with some flights conducted up to 4 hr from solar noon.

Prior to the start of each flight, an image containing a MAPIR Camera Reflectance Calibration Ground Target Package was collected. The reflectance values of individual images were calibrated against the target values using MAPIR Camera Control software. Orthomosaics and digital elevation models were generated from the calibrated images for each warren‐control plot pair in Metashape Professional version 1.6.2. The resulting orthomosaics were converted into raster files, the red and near infra‐red (NIR) bands separated and normalized difference vegetation index (NDVI) values were calculated for each pixel using the formula:

$$\text{NDVI}=\left(\text{NIR}‐\text{RED}\right)/\left(\text{NIR}+\text{RED}\right)$$

A new raster file containing the NDVI values for each pixel was generated for each flight.

A k‐means unsupervised classification was applied to the NDVI raster files to separate vegetated and nonvegetated pixels using the unsuperClass function in RStoolbox version 0.2.6 for R Statistical Software version 3.6.2 (Leutner et al., 2019). Manual inspection of the resulting geometries confirmed that the classification was highly accurate at assigning pixels containing green vegetation to the correct class. Some grass clumps were incorrectly assigned to the nonvegetated class, but as these were in a dormant state at the time of image collection due to drought conditions (i.e., the above‐ground structures were not photosynthesizing), we did not attempt to correct this. Geometries containing only the vegetated pixels were generated for each flight and these were used for assessing the difference in the NDVI values between warren and control plots.

### 2.6 Statistical analyses

We used nonmetric multidimensional scaling (nMDS) with significance assessed using permutational multivariate analysis of variance (vegan package for R version 2.5‐6; Oksanen et al., 2019) to assess overall differences between either the warren microsites and controls (soil variables) or the warrens and controls (vegetation cover and productivity variables) and to identify which variables were most important for structuring the soils and plant communities. For each of the input variables that contributed significantly to structuring the soils and vegetation communities, and for ground cover and scat abundance, we assessed differences between the warren microsites and controls (soil variables) or the warrens and controls (vegetation, ground cover and scat abundance variables) using generalized linear mixed‐effects models, from the lmer4 package version 1.1‐21 for R Statistical Software version 3.6.2 (Bates et al., 2015; R Core Team, 2019). We used a Poisson distribution for plant species richness and a Gaussian distribution for all soil properties and vegetation and ground cover. A negative binomial distribution was used for scat abundance because the data contained a high proportion of true zeros. Vegetation cover was also analyzed separately for the following functional types: large shrubs (shrub species > 1 m tall as adults, including small individuals of these species), small shrubs (shrub species < 1 m tall as adults), grasses, native species, non‐native species and dead vegetation. Differences in the percent cover of plant species that were significantly correlated with either the warren or control plots (see below), and the five most frequently recorded species at each property, were also tested using generalized linear mixed‐effects models.

We used correlation indices to identify plant species preferences for groups of sites (De Caceres & Legendre, 2009). We calculated an abundance‐based *phi* coefficient of association, using the “multipatt” function from the “indicspecies” package for R version 1.7.9 (De Caceres & Legendre, 2009) to test for species that are associated with either warrens or controls. We examined the effect of the warrens on plant species composition using nonmetric multidimensional scaling (nMDS) and assessed significance using permutational multivariate analysis of variance (vegan package for R version 2.5‐6; Oksanen et al., 2019).

We conducted separate models for each site (i.e., Yookamurra, Matuwa and Faure) as, given their large geographical separation, we assumed that both soils and vegetation will vary. We used the mean values for each microsite or control for the soil properties, and the mean values for each warren or control for vegetation or ground percent cover. Plot‐pair was used as a random effect in all models to account for the nonindependence of the samples among the microsites (soil variables) and the paired nature of our warren and control plots. Habitat, and the interaction between habitat and microsite or plot, were included as explanatory variables in all models. Data were log‐transformed where necessary to meet the model assumptions. Although percent data (i.e., moisture, carbon and percent cover variables) are often arcsine transformed, we found this inadequately transformed our data so used a log transformation after Warton and Hui (2011).

## 3 RESULTS

### 3.1 Soil properties and ground cover

#### 3.1.1 Soil properties

We found clear distinctions between the soils of the warren and control microsites, and these differences were significant at all three sites (based on nMDS analyses; Faure *r* ^2^ = 0.294, *p* = 0.001; Matuwa *r* ^2^ = 0.295, *p* = 0.001; Yookamurra *r* ^2^ = 0.175, *p* = 0.001). All of the soil variables we assessed were significant contributors to the models for at least two of the three sites. Most soil variables differed between the warrens (particularly the entrance and spoil microsites) and the controls in at least one habitat at each site (Figure 2). Although we detected variation in the magnitude of these differences across our three sites, the direction of change was generally consistent, even when the difference was not significant. The warren microsites and the controls varied from each other in a generally consistent pattern: entrances and spoils were often significantly different from the matrix and control microsites, but these pairs were only occasionally significantly different from each other (e.g., matrix and controls rarely differed).

Moisture levels at Faure were very low (only 10% of readings returned a value > 0) and did not differ between the microsites on warrens and the controls (Figure 2, Table A1). At Matuwa, spoils had significantly higher moisture levels than the controls and the other warren microsites in the shrub but were only significantly higher than the matrix microsite in the spinifex habitat (Figure 2, Table A1). Moisture levels were significantly higher in the entrances compared to the control and matrix microsites in both habitats at Yookamurra (Figure 2, Table A1).

Entrances and spoils generally had lower soil compaction than matrix microsites and the controls (Figure 2, Table A1). This was significant between entrances and both controls and the matrix microsites in all habitats and sites except the mallee habitat at Yookamurra (Figure 2). Ammonium nitrogen, nitrate nitrogen, potassium and sulfur were significantly higher on the entrances and spoils compared to the controls and the matrix microsites in most habitats and sites (Figure 2, Table A1). Phosphorus and carbon were significantly lower on the entrances and spoils compared to the controls and the matrix microsites in most habitats and sites (Figure 2) except in the myoporum habitat at Yookamurra where phosphorus was higher in the spoils compared to the controls (Figure 2, Table A1).

#### 3.1.2 Ground cover

The amount of ground disturbed by animals was significantly higher on boodie warrens at all three sites (Table A2). Boodie warrens also had more bare ground and this was significant in all habitats except the spinifex at Matuwa (Table A2). Warrens generally had less leaf litter, but this was significant only in the mallee at Yookamurra (*t*_14.8_ = 3.72, *p* = 0.01). Cryptogamic crust was only rarely recorded at Faure and was uncommon in the spinifex habitat at Matuwa. In the shrub and myoporum habitats at Matuwa and Yookamurra, cryptogamic crust cover was significantly lower on the warrens compared to the controls (Matuwa *t*_19.3_ = 4.79, *p* < 0.001; Yookamurra *t*_44.4_ = 4.1, *p* = 0.001; Table A2).

### 3.2 Scat abundance

Total scat and bettong scat abundance were highly correlated at all three sites. Warrens at Matuwa and Faure had significantly more bettong scat than control plots (Matuwa shrub *t*_19.3_ = −5.29, *p* < 0.001; Matuwa spinifex *t*_19.3_ = −3.99, *p* = 0.004; Faure *t*_16.1_ = −6.91, *p* < 0.001; Table A3). At Matuwa, rabbit (*Oryctolagus cuniculus*) scat was recorded at every warren and was significantly more abundant on the warrens in both habitats (shrub *t*_19.3_ = −8.83, *p* < 0.001; spinifex *t*_19.3_ = −4.08, *p* = 0.003; Table A3). We occasionally recorded the scat of other species; banded hare‐wallaby (*Lagostrophus fasciatus*) and Shark Bay bandicoot (*Perameles bougainville*) at Faure; mala (*Lagorchestes hirsutus*), golden bandicoot (*Isoodon auratus*) and common brushtail possum (*Trichosurus vulpecula*) at Matuwa; and eastern grey kangaroo (*Macropus giganteus*), greater bilby (*Macrotis lagotis*), short‐beaked echidna (*Tachyglossus aculeatus*) and common brushtail possum at Yookamurra. The abundance of scat from these species did not differ significantly between the warren and control plots.

### 3.3 Warren use by other vertebrates

While recording other data we observed several species using the warrens. At Yookamurra, two juvenile numbats (*Myrmecobius fasciatus*) were seen repeatedly entering and exiting apparently little used entrances of a large, active warren, and basking on the spoil piles. The numbats were observed on a number of occasions spanning several weeks. Dead brushtail possums were recorded in the burrow entrances of a number of warrens at Yookamurra. At Matuwa, we observed three species of reptiles, *Varanus gouldii, Eremiascincus richardsonii* and *Furina ornata*, exiting or entering the burrows.

### 3.4 Vegetation properties

The nMDS analyses showed that, overall, vegetation properties were similar on boodie warrens and controls at all three sites. All of the vegetation variables we assessed were significant contributors to the models for at least two of the three sites.

#### 3.4.1 Species richness

**FIGURE 3:**
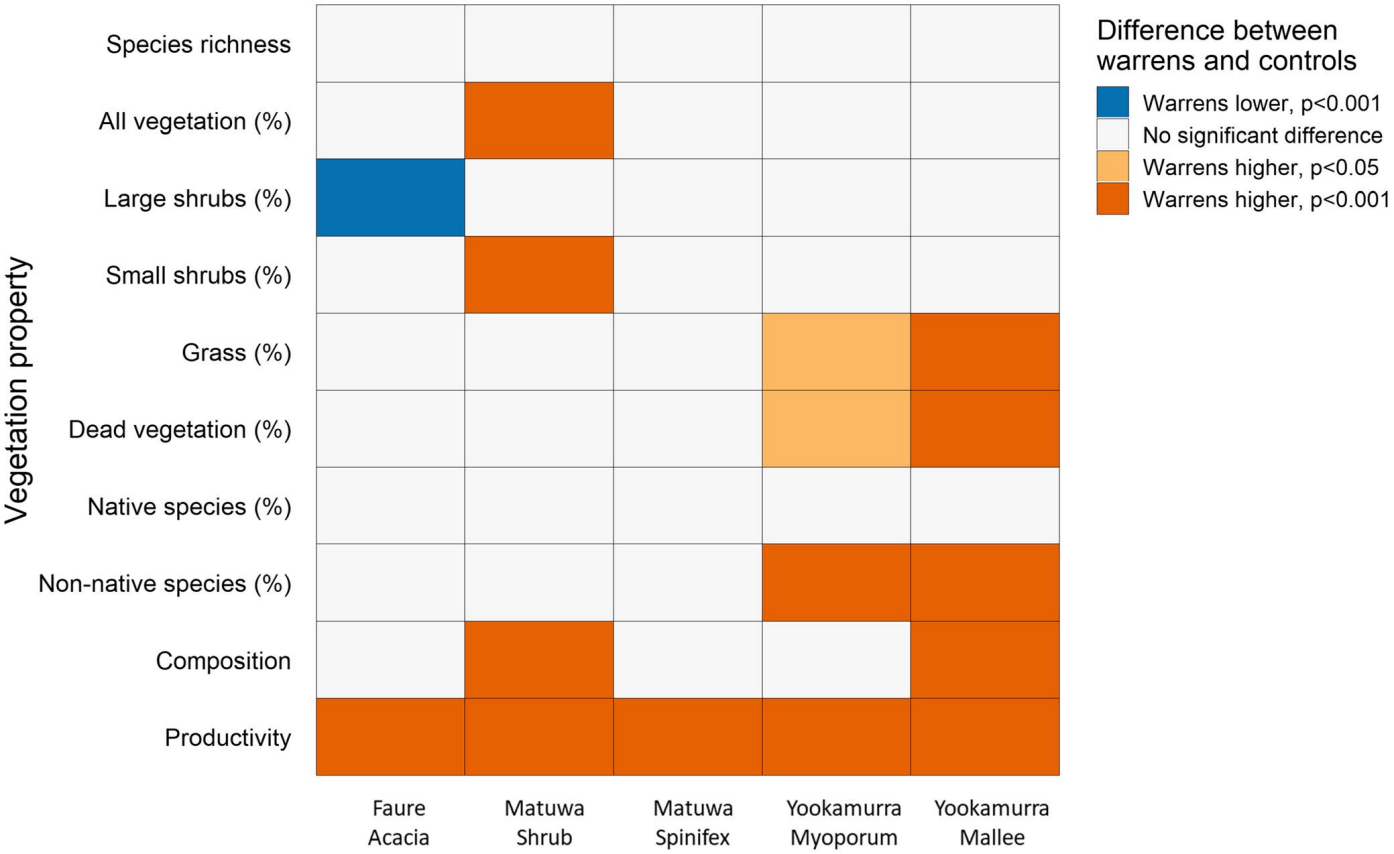
There were few differences in vegetation species richness, percent cover or composition between boodie warrens and undisturbed control plots. Plot shows the results (*p* values) of generalized linear mixed‐effects models comparing vegetation properties between warrens and controls

Vegetation species richness did not differ between warrens and controls at any of the three sites (Figure 3, Table A4). We did not record any non‐native species at Matuwa so we did not assess differences between native and non‐native species for that site. Neither native nor non‐native species richness differed between warrens and controls at Faure or Yookamurra (Table A4).

#### 3.4.2 Cover

There were few differences in the mean percent cover of most plant groups between warrens and controls at any of the three sites (Figure 3, Table A5). Exceptions to this were significantly higher total vegetation (*t*_19.3_ = −3.59, *p* = 0.009) and small shrub (*t*_19.3_ = −3.59, *p* = 0.004) cover on the warrens in the shrub habitat at Matuwa, higher cover of non‐native species on the warrens in both habitats at Yookamurra (myoporum *t*_22.2_ = −3.25, *p* = 0.017; mallee *t*_22.2_ = −3.85, *p* = 0.004), higher cover of grasses (*t*_22.2_ = −4.12, *p* = 0.002) and dead vegetation (*t*_22.2_ = −3.38, *p* = 0.013) on the warrens in the mallee habitat at Yookamurra and lower cover of large shrubs (*t*_16.1_ = 2.22, *p* = 0.041) on the warrens at Faure (Figure 3, Table A5).

#### 3.4.3 Correlation indices & individual species responses

**FIGURE 4:**
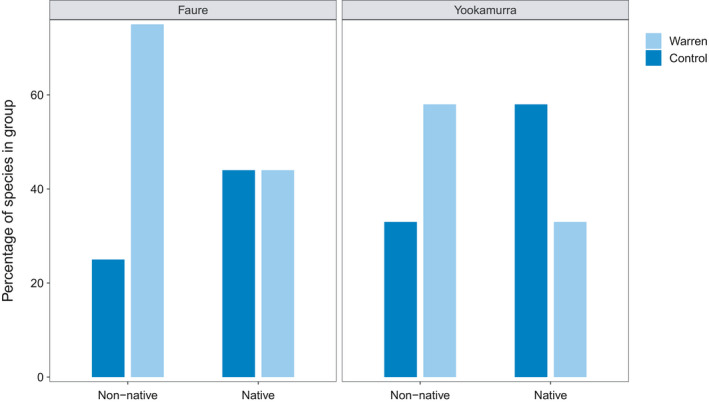
At both Faure and Yookamurra, more non‐native plant species were positively associated with (i.e., more abundant and found more commonly at) boodie warrens than control plots. Percentages for each group do not always sum to 100 because some species were associated with both warrens and controls. Data from the two habitat types at Yookamurra are combined

We found substantial differences in the response of native and non‐native plant species to warren presence at Faure and Yookamurra (no non‐native species were recorded at Matuwa). A greater proportion of non‐native species were positively associated with warrens compared to controls at both Faure (75% c.f. 25%) and Yookamurra (58% c.f. 33%; Figure 4). In contrast, native species did not respond to warren presence at Faure (the same proportions of native species (44%) were positively associated with warrens and controls), and a greater proportion of native species were positively associated with controls rather than warrens at Yookamurra (58% c.f. 33%; Figure 4).

Six species, three of which were non‐native, were significantly associated with warrens (i.e., were more abundant and found more commonly on warrens) across the three sites*. Herniaria cinerea, Schismus barbatus,* and *Sclerolaena obliquicuspis* were found more commonly on warrens in the myoporum habitat at Yookamurra, *Senna artemisioides* and an unidentified grass were significantly associated with warrens in the shrub habitat at Matuwa, and *Sisymbrium orientale* was found more commonly on warrens at Faure (Table A6). Native species were more likely to be significantly associated with controls. At Matuwa three species, two native acacias and an unknown grass sp., were found more commonly on the controls and, at Yookamurra, eight species, all native, were found more commonly on the controls (Table A6). No species were significantly correlated with the controls at Faure (Table A6).

Four species, one from Faure and three from Yookamurra, had significantly higher cover on the warrens compared to the controls: These included three annual, non‐native species and one native, perennial shrub (Table A6). All four species are known to respond positively to disturbance. Five species, one each from Faure and Matuwa and three from Yookamurra, had significantly higher cover on the control plots. All five species were native species: two perennial shrubs, two annual herbs, and an unidentified grass (Table A6).

#### 3.4.4 Species composition

There were significant differences in species composition between warrens and controls in the mallee habitat at Yookamurra (*r* ^2^ = 0.16, *p* = 0.05) and the shrub habitat at Matuwa (*r* ^2^ = 0.16, *p* = 0.04; Figure 3). Species composition did not differ in the other habitat types at Matuwa and Yookamurra or at Faure. There were nine species that were recorded only on the warrens and five that were recorded only on the controls at Faure (Table A7). At Matuwa, seven species were recorded only on the warrens and nine only on the controls (Table A7). There were fourteen species recorded only on the warrens and seventeen species recorded only on the controls at Yookamurra (Table A7).

#### 3.4.5 Productivity

Plant productivity was significantly higher on boodie warrens compared to control plots at all three sites (Figure 3, Table A8). When a buffer zone around the edge of the warren was added, to encompass any potential effects of underground structures extending beyond the disturbance visible at the surface, the difference between the mean NDVI values of warrens and control plots remained significant for Faure (*t*_11270_ = −7.05, *p* < 0.001) and Yookamurra (*t*_62060_ = 9.73, *p* < 0.001) but became marginally nonsignificant at Matuwa. However, the mean NDVI values of warren and control plots were all below zero (indicating most of the vegetation was not photosynthetically active) and differed by less than 0.01 in all cases (Table A8). The NDVI values of vegetated pixels increased significantly with distance from the center of warrens at Yookamurra (*t*_31440_ = −8.91, *p* < 0.001) but not at either Faure or Matuwa.

### 3.5 Warren distribution, size, and activity level

Warren density was far higher at Faure (5.65 warrens/ha) than at either Yookamurra (0.3 warrens/ha) or Matuwa (0.1 warrens/ha; Table 2). The proportion of warrens with at least one active entrance was also highest at Faure (Table 2). Warrens at Matuwa were significantly larger and had significantly more entrances and significantly more active entrances than either Faure or Yookamurra (Table 2). Warrens at Faure were the smallest, with significantly fewer total entrances than warrens at Yookamurra (Table 2).

**TABLE 2:** The density, size, and activity levels of boodie warrens at three sites

	Faure	Matuwa	Yookamurra	*p* value
Warrens ha^−1^	5.65	0.1	0.3	
Proportion of warrens active	95%	57%	79%	
Mean size (m^2^)	133.88 (17.12)	452.98 (68.36)	186.68 (21.1)	M > Y, F (*p* < .01)
Area covered by warrens	7.5%	0.45%	0.5%	
Mean number entrances	3.11 (0.51)	13.58 (2.02)	7.05 (0.66)	M > Y, F (*p* < .01); Y > F (*p* < .01)
Mean number active entrances	2.64 (0.47)	9.37 (1.63)	2.46 (0.35)	M > Y, F (*p* < .01)

## 4 DISCUSSION

Boodie warrens altered soil properties, but had only limited impacts on vegetation communities, across a range of habitats and sites. Although boodie warrens varied in size and density according to the substrate available at each study site, they tended to have similar effects on soils, ground cover, vegetation, and novel ecosystem elements. Some plant groups in some habitats, such as small shrubs in the shrub habitat at Matuwa, had higher cover on the warrens, and several native vertebrate species, other than boodies, were recorded using the warrens. However, novel ecosystem elements, such as non‐native plant species and rabbits, also benefited from the conditions found on boodie warrens, indicating that boodies may contribute to ecosystem changes.

### 4.1 Burrowing mammals alter soil properties

Overall, we found that soil properties and ground cover often differed between warrens and controls. Unsurprisingly, boodie warrens had significantly more bare and disturbed ground, and tended to have less leaf litter and cryptogamic crust. The magnitude of the differences in the soil properties across our three sites varied but the direction of change was generally consistent. Two of the microsites on boodie warrens, entrances and spoils, tended to have higher moisture and lower compaction levels than controls. Of the soil nutrients we assessed, nitrogen, sulfur and potassium levels tended to be higher, and phosphorus and carbon levels lower, on entrances and spoils compared to the controls. These results are largely consistent with those recorded for other burrowing mammals (Hagenah et al., 2013, e.g., Kerley et al., 2004, Wesche et al., 2007). However, previous studies of relict boodie warrens have recorded higher levels of phosphorus and carbon on warrens (Chapman, 2015; Noble et al., 2007). In contrast to the relatively inactive relict warrens, the high levels of soil turnover on the active warrens in our study most likely reduced soil carbon through increased decomposition rates or the deposition of subsurface soil containing little organic matter on the surface (Kurek et al., 2013; Sherrod & Seastedt, 2001).

We found that matrix microsites on the warrens were largely similar to the controls. This suggests that, rather than selecting warren locations based on their compaction, moisture, or nutrient levels, boodies cause the soil property differences we observed. Compaction was most likely reduced through the reworking of the soil during burrow construction. Less compact soils may subsequently lead to higher moisture levels through increased infiltration rates (Fleming et al., 2014). Increased soil nutrient levels on burrows and warrens have previously been attributed to the accumulation of urine, feces and decomposing nesting material (Hagenah et al., 2013; Moorhead et al., 1988). We found that boodie scat was 2–14 times more abundant on warrens, and primarily concentrated around entrances and spoils, where nutrient concentrations were also higher. Boodies also accumulate nesting material within their warrens (Stodart, 1966); its decomposition may be enriching the sub‐surface soil which is later brought to the surface. Through their deposition of organic material and their construction activities, boodies are significantly changing the compaction, moisture, and nutrient contents of the soils on their warrens.

### 4.2 Vegetation communities on warrens were similar to undisturbed sites

Many Australian soils are poor in nutrients, especially phosphorus and nitrogen, and this, along with a lack of water, is a primary limiting factor for plants (Smith & Morton, 1990). The higher nutrient and moisture concentrations on boodie warrens may make them favorable sites for vegetation, but, surprisingly, we found that there were few differences between the plant communities on warren and control plots. Many other studies comparing plant communities between mammal burrows and control sites have found significant differences in vegetative cover (e.g., Haussmann et al., 2018; Parsons et al., 2016; Wheeler & Hik, 2013), species richness (e.g., Davidson & Lightfoot, 2008; Hagenah et al., 2013), and composition (e.g., Bryce et al., 2013; Davidson & Lightfoot, 2006; Wesche et al., 2007). Noble et al. (2007) found that uninhabited, relict boodie warrens had a higher cover of palatable grasses and forbs, indicating that boodie warrens do have the potential to affect vegetation communities. At our study sites, other factors, such as herbivory or weather conditions, may have stronger influences on vegetation communities than the soil changes brought about by boodie warren construction. Additionally, Australian arid and semi‐arid zone vegetation communities are slow growing, and more time may be required for more pronounced differences to become apparent.

Increased grazing pressure on the plants growing on the warrens may be off‐setting any positive effects of the increased moisture and nutrients we recorded on boodie warrens. The net effect of boodies, and other reintroduced mammals, on seedling abundance has been shown to be negative due to the effects of herbivory despite the apparent benefits of their foraging diggings on seedling abundance (Verdon et al., 2016). Plants growing on boodie warrens contain higher concentrations of nutrients (Chapman, 2015) and the burrows of steppe marmots (*Marmota bobak*) support a greater abundance of palatable species (Valkó et al., 2021). Nutrient‐rich, palatable foliage attracts herbivores and can lead to lower overall plant biomass even though plants can benefit from increased soil nutrients (van der Waal et al., 2016). Further experimental work, incorporating exclusion devices on inhabited warrens, could help to disentangle the engineering and trophic effects of boodies and boodie warrens on vegetation.

Environmental conditions, particularly rainfall, are likely to affect the impact of boodie warrens on vegetation. Moorhead et al. (1988) found, for example, that the cover of spring annuals was significantly higher on banner‐tailed kangaroo rat (*Dipodomys spectabilis*) mounds but only in years of sufficient rainfall. Arid zone Australia is characterized by highly variable rainfall resulting in distinct pulses in plant productivity. It is only in wet years that substantial amounts of annual vegetation are present and perennial plants establish (Morton et al., 2011). Our research was carried out in a time of extreme drought at all three sites, which is likely to have reduced vegetative responses and may have exacerbated trophic impacts. Long‐term monitoring of vegetation on and off boodie warrens may be the only way to obtain a complete understanding of their effect on plant communities.

### 4.3 Boodie warrens benefit novel ecosystem elements

We found that non‐native plant species appeared to benefit from the conditions found on boodie warrens more than native plant species. This may be because the amount of disturbed ground was significantly higher on the warrens and many non‐native plants are adapted to disturbance (Liebman et al., 2001). Alternatively, the increased moisture and nutrient levels on the warrens may be more important for non‐native species. For example, Ward's weed (*Carrichtera annua*), which was significantly more abundant on warrens at Yookamurra, will not germinate when soil moisture is very low (Facelli & Chesson, 2008). Browsing pressure on palatable, native species on the warrens could have decreased competition, allowing unpalatable, non‐native species to increase. Preferential browsing by black‐tailed prairie‐dogs (*Cynomys ludovicianus*) has been shown to increase the cover of non‐native forbs at four times the rate of native forbs (Beals et al., 2014). Because boodie warrens appear to be favorable locations for non‐native plant species, in areas where there are species of management concern, it may be prudent to control or eradicate these prior to translocating boodies. In situations where this is not possible, boodie warrens could be used as focal points for control of problem species post‐release.

At Matuwa, boodie warrens are used extensively by introduced rabbits. Boodie warrens are thought to have facilitated the rapid spread of rabbits throughout arid Australia in the late 19th and early 20th centuries (Parer & Libke, 1985); an example of how native ecosystem engineers can have detrimental effects under altered environmental conditions. While boodies appear to be largely unaffected by the presence of rabbits, more sensitive species that compete directly for food resources may be adversely affected. If translocations of boodies are planned to areas supporting rabbits, it is probably best to eradicate them prior to releasing boodies, to prevent further expansion of the rabbit population.

Boodie warrens are also used by native fauna (Read et al., 2008) and our study added to the number of native species known to benefit from their presence. We opportunistically recorded seven species of native mammals and reptiles sheltering within the warrens and recorded the scats of a further seven native mammals on the warrens. Our observation of a numbat using an active warren to raise its young shows that boodie warrens can provide key breeding resources to other threatened species. In areas where breeding resources, such as tree hollows or burrows, are limited, the prior translocation of boodies or other burrowing species may increase the chances of other species successfully establishing.

### 4.4 Soil substrate influences warren characteristics

The translocated boodie populations in our study constructed large, complex warrens on the harder soils at Yookamurra and Matuwa (myoporum and shrub habitats) and smaller, simpler warrens in the softer sands (mallee, spinifex and acacia habitats) at all three sites. Warrens constructed by extant boodie populations are most commonly described as large structures created by boodies burrowing into or under caprock or calcrete lenses (Burbidge et al., 2007; Noble et al., 2007; Sander et al., 1997). In the coastal sand on Bernier and Dorre Islands, however, boodies also construct smaller, simpler warrens under shrubs and tussock grasses (Ride et al., 1962). It is likely that boodies once constructed warrens in softer sands throughout their former range, but these warrens would have disappeared not long after boodies were extirpated. Large warrens, such as those that have persisted long after boodies disappeared, appear to be restricted to locations where a calcrete layer or caprock provides long‐term structural integrity.

Boodie warren density in our study was likely driven by substrate characteristics, though population density may have also been influential. At Matuwa, and to some extent Yookamurra, the calcrete layer prevents the construction of warrens in many areas but, where opportunities to dig under it exist, it provides structural integrity and allows warrens to expand into substantial structures. As a result, Matuwa, where the calcrete layer is particularly thick and close to the surface, has the lowest warren density but the warrens there are significantly larger and have significantly more burrow entrances than the warrens at Yookamurra and Faure. A similar pattern of warren density, size and activity level has been recorded in southern hairy‐nosed wombats (Walker et al., 2007). Future boodie translocations should, therefore, consider substrate characteristics when making predictions about warren and population densities.

The impact of warren sizes and density on boodie population structure and dynamics will contribute to shaping the impact that boodies have on the wider landscape. Boodies tend to stay within a few hundred meters of their warren (Finlayson & Moseby, 2004; Short & Turner, 1999). Where relatively few, large warrens occur, as we observed in the shrub habitat at Matuwa, the wider ecosystem impacts of boodies, that is, through herbivory or foraging digs, may be relatively concentrated. In areas where many, small warrens are constructed, such as we recorded at Faure, more diffuse ecosystem impacts may occur. An understanding of how soil substrate influences potential boodie warren size and density could allow translocation practitioners to predict the overall ecosystem effects of translocating boodies. Although it may be difficult to conduct given the current confinement of boodies to islands and fenced areas, further work is still required to determine the link between warren density and the landscape‐scale impacts of boodies.

If there is a consistent relationship between boodie warren density and population size it may be possible to use the presence of relict warrens to estimate historic population densities. However, because warrens constructed in sands are likely to degrade quickly, the density of relict warrens will most likely underrepresent historic warren densities. The warren densities we observed at Matuwa and Yookamurra are similar to those recorded in studies of relict warrens (Burbidge et al., 2007; Noble et al., 2007) suggesting Matuwa and Yookamurra boodie population densities may be lower than, or similar to, historic densities. At Faure, the population density is more than ten times that of Matuwa and Yookamurra, possibly because the sandy soils allow unconstrained warren construction. Although there is concern that the population density of several confined boodie populations is too high, early European reports suggest that boodies previously occurred in very high densities (Abbott, 2008) and we hypothesize that current population densities at our three sites are likely to fall within the range of densities previously exhibited by boodies in different habitat and soil types.

The extreme drought conditions operating at all three sites during our study may have prevented us detecting the full range of effects on vegetation communities. However, with increased temperatures and lower, but more variable, rainfall predicted under climate change (CSIRO & Bureau of Meteorology, 2015), drought may most closely represent future prevailing weather conditions. Since climatic conditions now and in the future are likely to differ from past conditions, translocating boodies may never result in the restoration of historic vegetation communities. However, as temperatures rise, warrens may become increasingly important for the persistence of other fauna at a site (Catano & Stout, 2015). While translocated boodies may not perfectly replicate the roles and effects of historic populations, they are likely to substantially alter the locations in which they are released.

## 5 CONCLUSIONS

Our study shows that, through the construction of substantially sized, numerous, and sometimes long‐lived warrens, translocated boodie populations play an important role in structuring ecosystems. Boodie warrens consistently modify soils across a range of habitats and sites by increasing soil moisture and many soil nutrients, and decreasing compaction. Although we did not find substantial impacts on vegetation communities, the higher cover of some plant groups and altered species compositions on warrens in two habitats indicate that warrens do have some effect. Due to the absence of data from prior to boodie population declines, it is impossible to know whether the effects we detected represent the restoration of boodies’ historic roles or, conversely, novel impacts and interactions. Regardless, boodies should be considered ecosystem engineers with the potential to alter soil resources for both plants and animals.

## CONFLICT OF INTEREST

None declared.

## AUTHOR CONTRIBUTIONS

**Bryony J. Palmer:** Conceptualization (lead); formal analysis (lead); investigation (lead); methodology (lead); writing – original draft (lead); writing – review and editing (equal). **Leonie E. Valentine:** Conceptualization (supporting); methodology (supporting); writing – review and editing (equal). **Cheryl A. Lohr:** Formal analysis (supporting); writing – review and editing (equal). **Gergana N. Daskalova:** Investigation (supporting); methodology (supporting); writing – review and editing (equal). **Richard J. Hobbs:** Conceptualization (supporting); methodology (supporting); writing – review and editing (equal).

## Data Availability

Data used in this study are available for download on Dryad (https://doi.org/10.5061/dryad.w3r2280pp).

## Appendix A Boodie warren effects on soils and vegetation

**TABLE A1:** The mean values of soil properties at four microsites on boodie warrens at three sites, Yookamurra, Matuwa, and Faure

Microsite	Control	Entrance	Matrix	Spoil	Control	Entrance	Matrix	Spoil
Yookamurra	Mallee				Myoporum			
Moisture %	3.4 ± 0.33	5.9 ± 0.22	4.2 ± 0.28	5.1 ± 0.39	3.9 ± 0.29	5.2 ± 0.34	4 ± 0.29	4.7 ± 0.30
Compaction kg/cm^2^	6.8 ± 1.55	5.0 ± 0.60	7.7 ± 1.40	4.4 ± 0.43	15.9 ± 0.58	7.8 ± 0.74	13.9 ± 1.29	7.2 ± 0.70
NH_4_ nitrogen mg/kg	3.6 ± 1.01	10.6 ± 5.71	2.8 ± 1.01	3.0 ± 0.47	2.3 ± 0.40	14.4 ± 4.01	2.3 ± 0.16	3.3 ± 0.68
NO_3_ nitrogen mg/kg	12.8 ± 4.81	20.0 ± 7.16	4.8 ± 2.40	17.1 ± 3.50	1.5 ± 0.12	41.2 ± 11.59	2.8 ± 1.03	20.3 ± 6.33
Phosphorus mg/kg	28.7 ± 4.97	15.5 ± 1.97	29.1 ± 2.76	15.8 ± 1.89	7.1 ± 0.88	10.5 ± 0.68	22.5 ± 3.48	14.1 ± 1.68
Potassium mg/kg	414.8 ± 24.40	828.5 ± 83.36	536.9 ± 41.89	767.6 ± 62.54	405.9 ± 20.74	1,165 ± 156.13	590.7 ± 55.62	882.6 ± 47.62
Sulfur mg/kg	46.4 ± 17.89	45.1 ± 6.21	8.9 ± 1.40	49.7 ± 13.28	2.7 ± 0.10	37.3 ± 8.35	3.9 ± 0.70	17.8 ± 4.14
Carbon %	3.4 ± 0.41	1.6 ± 0.17	2.4 ± 0.30	1.6 ± 0.15	1.1 ± 0.06	0.9 ± 0.09	1.3 ± 0.17	0.8 ± 0.10
Conductivity dS/m	0.4 ± 0.06	0.6 ± 0.07	0.2 ± 0.04	0.5 ± 0.08	0.1 ± 0	0.5 ± 0.09	0.1 ± 0.01	0.3 ± 0.04
pH (H_2_O)	7.9 ± 0.08	8.2 ± 0.08	7.9 ± 0.05	8.2 ± 0.09	7.8 ± 0.02	8.2 ± 0.06	7.9 ± 0.03	8.1 ± 0.06
pH (CaCl_2_)	8.5 ± 0.05	9.3 ± 0.03	8.7 ± 0.02	9.2 ± 0.04	8.7 ± 0.02	9.1 ± 0.03	8.8 ± 0.02	9.2 ± 0.03
Matuwa	Spinifex				Shrub			
Moisture %	1.8 ± 0.20	1.8 ± 0.12	1.3 ± 0.15	2.2 ± 0.10	3.1 ± 0.24	3 ± 0.13	3.7 ± 0.22	4.1 ± 0.22
Compaction kg/cm^2^	8.9 ± 1.35	2.2 ± 0.49	5.6 ± 0.99	4.2 ± 0.46	14.2 ± 0.56	3.2 ± 0.51	8.3 ± 0.85	4.1 ± 0.45
NH_4_ nitrogen mg/kg	3.6 ± 0.34	48.4 ± 8.41	14.9 ± 3.38	38.2 ± 6.07	2.2 ± 0.59	15 ± 1.98	2 ± 0.19	5.2 ± 0.89
NO_3_ nitrogen mg/kg	4.0 ± 1.64	13.3 ± 3.34	2.1 ± 0.29	2.1 ± 0.52	4.2 ± 0.53	89.4 ± 42.42	6.7 ± 1.01	25 ± 11.95
Phosphorus mg/kg	5.6 ± 0.46	2.6 ± 0.36	4.2 ± 0.35	2.2 ± 0.23	7.2 ± 0.76	45.6 ± 13.98	20.7 ± 3.30	33.9 ± 15.02
Potassium mg/kg	121.9 ± 15.42	222.6 ± 25.99	125.5 ± 11.23	201.1 ± 13.92	212.4 ± 13.18	411 ± 45.25	256.2 ± 17.52	372.1 ± 46.63
Sulfur mg/kg	3.7 ± 0.51	14.4 ± 3.28	3.4 ± 0.30	4.5 ± 0.37	9.2 ± 2.30	459.5 ± 289.10	6.8 ± 1.51	357.8 ± 294.60
Carbon %	0.3 ± 0.03	0.2 ± 0.02	0.3 ± 0.03	0.1 ± 0.01	0.4 ± 0.03	0.7 ± 0.11	0.5 ± 0.05	0.4 ± 0.09
Conductivity dS/m	0 ± 0.03	0.1 ± 0.02	0 ± 0.03	0 ± 0.01	0.1 ± 0.03	0.5 ± 0.11	0.1 ± 0.05	0.3 ± 0.09
pH (H_2_O)	5.8 ± 0.09	6.8 ± 0.18	6.5 ± 0.12	7.0 ± 0.16	8.6 ± 0.07	8.4 ± 0.10	8.7 ± 0.05	8.6 ± 0.09
pH (CaCL_2_)	4.8 ± 0.09	6.2 ± 0.23	5.4 ± 0.18	6.1 ± 0.21	7.8 ± 0.07	7.8 ± 0.07	7.9 ± 0.04	7.9 ± 0.06
Faure	Acacia							
Moisture %	0 ± 0.01	0.1 ± 0.03	0.2 ± 0.21	0.1 ± 0.08				
Compaction kg/cm^2^	2.8 ± 0.15	1.4 ± 0.10	3.0 ± 0.30	2.8 ± 0.20				
NH_4_ nitrogen mg/kg	2.1 ± 0.42	12.4 ± 1.75	4.7 ± 1.79	6.8 ± 1.4				
NO_3_ nitrogen mg/kg	6.7 ± 1.21	32.9 ± 3.88	9.2 ± 1.91	12.7 ± 1.95				
Phosphorus mg/kg	9.6 ± 1.29	5.7 ± 0.58	9.1 ± 0.69	6.1 ± 0.48				
Potassium mg/kg	144.1 ± 15.43	239.7 ± 18.94	151.3 ± 10.23	187.1 ± 14.42				
Sulfur mg/kg	5.1 ± 0.96	18.1 ± 3.50	6.3 ± 1.46	4.7 ± 0.68				
Carbon %	0.5 ± 0.03	0.3 ± 0.03	0.6 ± 0.06	0.3 ± 0.03				
Conductivity dS/m	0.1 ± 0.01	0.2 ± 0.02	0.1 ± 0.01	0.1 ± 0.01				
pH (H_2_O)	7.7 ± 0.14	7.7 ± 0.12	7.7 ± 0.09	7.6 ± 0.13				
pH (CaCL_2_)	6.9 ± 0.14	7.0 ± 0.12	6.8 ± 0.10	6.8 ± 0.12				

Note

Data presented are the mean ± standard error.

**TABLE A2:** The mean percent cover of ground variables on boodie warrens and paired controls at three sites, Yookamurra, Matuwa, and Faure

Microsite	Warren	Control	Warren	Control
Yookamurra	Mallee		Myoporum	
Disturbed ground	58.96 ± 5.51	10.41 ± 2.18	55.02 ± 6.48	8.88 ± 1.83
Bare ground	49.15 ± 3.92	34.67 ± 4.04	40.95 ± 3.08	15.45 ± 1.24
Leaf litter	23.76 ± 3.91	43.95 ± 5.80	16.82 ± 2.05	17.18 ± 2.41
Cryptogamic crust	20.13 ± 3.74	17.25 ± 6.87	32.38 ± 3.48	58.53 ± 2.86
Matuwa	Spinifex		Shrub	
Disturbed ground	34.95 ± 2.37	1.33 ± 0.55	41.89 ± 6.01	0.95 ± 0.44
Bare ground	61.84 ± 5.21	42.17 ± 6.29	77.24 ± 2.32	62.54 ± 4.25
Leaf litter	12.37 ± 2.42	14.79 ± 3.05	13.71 ± 1.68	11.10 ± 1.79
Cryptogamic crust	0.45 ± 0.38	3.41 ± 2.86	3.65 ± 0.82	23.28 ± 4.13
Faure	Acacia			
Disturbed ground	55.75 ± 2.35	4.15 ± 1.32		
Bare ground	46.48 ± 3.44	34.85 ± 5.20		
Leaf litter	21.89 ± 1.92	25.78 ± 3.29		
Cryptogamic crust	0.02 ± 0.02	0.14 ± 0.08		

Note

Data presented are the mean ± standard error.

**TABLE A3:** The mean abundance of scat recorded at boodie warrens and paired controls at three sites, Yookamurra, Matuwa, and Faure

Microsite	Warren	Control	Warren	Control
Yookamurra	Mallee		Myoporum	
All scat	2.27 ± 0.64	2.44 ± 0.61	4.89 ± 0.63	4.01 ± 0.65
Boodie	2.81 ± 0.60	2.55 ± 0.44	4.73 ± 0.54	3.05 ± 0.32
Matuwa	Spinifex		Shrub	
All scat	4.44 ± 0.61	0.48 ± 0.09	10.03 ± 0.89	1.05 ± 0.34
Boodie	5.07 ± 0.81	1.48 ± 0.31	4.61 ± 0.81	0.92 ± 0.15
Rabbit	4.76 ± 0.54	1.00 ± 0.56	8.58 ± 0.79	1.87 ± 0.36
Faure	Acacia			
All scat	5.21 ± 0.54	2.05 ± 0.39		
Boodie	4.99 ± 0.52	1.35 ± 0.25		

Note

Data presented are the mean ± standard error.

**TABLE A4:** The mean species richness of plants growing on boodie warrens and control plots at three sites, Yookamurra, Matuwa, and Faure

Microsite	Warren	Control	Warren	Control
Yookamurra	Mallee		Myoporum	
All	6.2 ± 0.68	5.3 ± 0.80	12.1 ± 1.06	13.3 ± 1.62
Native	5.2 ± 0.63	4.7 ± 0.68	10.1 ± 0.74	11.3 ± 1.26
Non‐native	1.0 ± 0.15	0.6 ± 0.16	2.0 ± 0.45	2.0 ± 0.47
Matuwa	Spinifex		Shrub	
All	2.8 ± 0.56	3.5 ± 0.46	5.4 ± 0.36	5.4 ± 0.35
Native	NA	NA	NA	NA
Non‐native	NA	NA	NA	NA
Faure	Acacia			
All	9.7 ± 0.50	9.1 ± 0.77		
Native	7.7 ± 0.49	7.6 ± 0.78		
Non‐native	2.0 ± 0.17	1.5 ± 0.19		

Note

Data presented are the mean ± standard error.

**TABLE A5:** The mean percent cover of vegetation groups on boodie warrens at three sites, Yookamurra, Matuwa, and Faure

Microsite	Warren	Control	Warren	Control
Yookamurra	Mallee		Myoporum	
All	0.07 ± 0.02	0.05 ± 0.01	0.10 ± 0.02	0.09 ± 0.03
Native	0.06 ± 0.02	0.05 ± 0.01	0.10 ± 0.02	0.12 ± 0.03
Non‐native	0.01 ± 0.004	0.001 ± 0.00	0.02 ± 0.01	0.01 ± 0.001
Big shrub	0.012 ± 0.01	0.004 ± 0.00	0.04 ± 0.01	0.06 ± 0.01
Little shrub	0.02 ± 0.01	0.02 ± 0.01	0.02 ± 0.01	0.02 ± 0.01
Grass	0.01 ± 0.004	0.002 ± 0.001	0.02 ± 0.01	0.01 ± 0.002
Herb	0.002 ± 0.00	0.001 ± 0.00	0.004 ± 0.00	0.004 ± 0.001
Dead	0.08 ± 0.03	0.03 ± 0.06	0.05 ± 0.01	0.02 ± 0.006
Matuwa	Spinifex		Shrub	
All	0.27 ± 0.05	0.41 ± 0.05	0.05 ± 0.01	0.02 ± 0.004
Native	NA	NA	NA	NA
Non‐native	NA	NA	NA	NA
Big shrub	0.01 ± 0.01	0.01 ± 0.002	0.01 ± 0.003	0.01 ± 0.002
Little shrub	0.0001 ± 0.0001	0.0004 ± 0.0004	0.02 ± 0.004	0.003 ± 0.001
Grass	0.24 ± 0.04	0.39 ± 0.04	0.01 ± 0.01	0.01 ± 0.003
Herb	NA	NA	NA	NA
Dead	0.02 ± 0.01	0.04 ± 0.02	0.12 ± 0.05	0.02 ± 0.004
Faure	Acacia			
All	0.31 ± 0.04	0.39 ± 0.05		
Native	0.29 ± 0.03	0.36 ± 0.04		
Non‐native	0.03 ± 0.01	0.04 ± 0.02		
Big shrub	0.21 ± 0.03	0.30 ± 0.04		
Little shrub	0.02 ± 0.01	0.02 ± 0.01		
Grass	0.02 ± 0.01	0.04 ± 0.02		
Herb	0.01 ± 0.002	0.003 ± 0.001		
Dead	0.15 ± 0.02	0.12 ± 0.03		

Note

Data presented are the mean ± standard error.

**TABLE A6:** The life attributes and percent cover of the five plant species most commonly recorded on, and species which were significantly associated with, either boodie warrens or controls at three sites, Yookamurra, Matuwa, and Faure.

Species	Life form	Origin	Palatability	Mean cover (standard error)		Mean cover (standard error)		*p* value
				Warren	Control	Warren	Control	
Yookamurra				Mallee		Myoporum		
*Asteridea athrixioides* ^c^	Annual	Native	Unknown	NA	NA	0.05 ± 0.05	0.2 ± 0.08	.08
*Austrostipa* sp.^a^	Perennial	Native	Palatable	0.33 ± 0.13	0.12 ± 0.08	0.64 ± 0.18	0.46 ± 0.05	.26
*Atriplex stipitata* ^a^	Perennial	Native	Unpalatable	3.53 ± 1.19	1.33 ± 2.91	7.57 ± 2.59	6.23 ± 0.29	**.03**
*Carrichtera annua* ^a^	Annual	Non‐native	Unpalatable	1.19 ± 0.57	2.91 ± 0.11	2.59 ± 0.52	0.29 ± 0.1	**.03**
*Geijera linearifolia* ^a^	Perennial	Native	Palatable	5.29 ± 3.53	2 ± 1.33	24.15 ± 7.57	17.27 ± 6.23	.4
*Goodenia pusilliflora* ^c^	Annual	Native	Palatable	NA	NA	NA	0.2 ± 0.08	**.03**
*Herniaria cinerea* ^b^	Annual	Non‐native	Unpalatable	NA	NA	0.2 ± 0.08	0.05 ± 0.05	.08
*Hyalosperma semisterile* ^c^	Annual	Native	Unknown	NA	NA	NA	0.2 ± 0.08	**.03**
*Olearia muelleri* ^c^	Perennial	Native	Unpalatable	0.7 ± 0.52	1.75 ± 1.03	NA	NA	**.04**
*Rhagodia crassifolia* ^c^	Perennial	Native	Palatable	1.15 ± 0.76	1.79 ± 0.84	NA	0.1 ± 0.1	.54
*Rytidosperma* sp.^a^	Perennial	Native	Palatable	NA	0.55 ± 0.55	0.38 ± 0.08	0.62 ± 0.15	.22
*Schismus barbatus* ^b^	Annual	Non‐native	Palatable	1.04 ± 0.96	NA	1.02 ± 0.51	0.15 ± 0.08	**.03**
*Sclerolaena obliquicuspis* ^b^	Perennial	Native	Low	NA	NA	0.35 ± 0.2	0.05 ± 0.05	.16
*Senna artemisioides* ^c^	Perennial	Native	Unpalatable	NA	0.4 ± 0.31	1.38 ± 0.77	2.47 ± 1.09	.17
Matuwa				Spinifex		Shrub		
*Acacia aneura var aneura* ^c^	Perennial	Native	Highly Palatable	0.63 ± 0.63	1.32 ± 0.77	NA	NA	.31
*Acacia* sp. 1^c^	Perennial	Native	Unknown	NA	NA	3.11 ± 1.98	4.83 ± 1.67	.18
*Aristida contorta* ^a^	Perennial	Native	Unpalatable	NA	NA	0.22 ± 0.22	0.95 ± 0.3	**.04**
*Grass* sp. 1^a^, ^b^	Unknown	Unknown	Unknown	NA	NA	1.87 ± 0.61	0.91 ± 0.44	.09
*Grass* sp. 4^a^, ^c^	Unknown	Unknown	Unknown	NA	NA	1.79 ± 0.6	0.44 ± 0.17	.88
*Ptilotus obovatus* ^a^	Perennial	Native	Palatable	NA	0.88 ± 0.88	11.1 ± 4.35	2.05 ± 0.56	.12
*Senna artemisioides* ^b^	Perennial	Native	Unpalatable	NA	NA	4.38 ± 1.73	2.36 ± 1.59	.08
*Triodia basedowii* ^a^	Perennial	Native	Unpalatable	34.55 ± 5.93	47.31 ± 2.73	NA	NA	.16
Faure				Acacia				
*Acacia ramulosa* ^a^	Perennial	Native	Palatable	22.86 ± 2.28	28.36 ± 2.38			.09
*Acacia tetragonophylla* ^a^	Perennial	Native	Palatable	14.64 ± 4.45	18.8 ± 6.9			.62
*Atriplex bunburyana* ^a^	Perennial	Native	Palatable	5.79 ± 2.28	5.75 ± 2.38			.25
*Cenchrus ciliaris* ^a^	Perennial	Non‐native	Palatable	3.05 ± 0.94	5.63 ± 2.37			.99
*Ptilotus obovatus* ^a^	Perennial	Native	Palatable	14.64 ± 4.45	18.8 ± 6.9			**.05**
*Sisymbrium orientale* ^b^	Annual	Non‐native	Low	1.27 ± 0.19	0.54 ± 0.07			**<.01**

^a^ Top five most commonly recorded species at site

^b^ Significantly associated with warrens

^c^ Significantly associated with controls

Significant differences at *p* < 0.05 are shown in bold text.

**TABLE A7:** Plant species recorded on only boodie warrens or controls at three sites, Yookamurra, Matuwa, and Faure

Yookamurra		Matuwa		Faure	
Warren	Control	Warren	Control	Warren	Control
*Crassula sieberiana*	*Asperula* sp.	*Acacia* sp. 3	*Acacia aneura var conifera*	*Acacia sclerosperma*	*Actinoble condensatum*
*Eremophila glabra*	*Bupleurum semicompositum*	*Acacia tetragonophylla*	*Acacia* sp. 1	*Asteraceae* sp. 1	*Aristida contorta*
*Lepidium sp*.	*Erodium crinitum*	*Grass* sp. 3	*Acacia* sp. 2	*Asteraceae* sp. 2	*Duperreya sp*.
*Olearia pimeleoides*	*Eucalyptus gracilis*	*Melaleuca interioris*	*Eremophila* sp. 2	*Austrostipa elegantissima*	*Euphorbia sp*.
*Rhagodia spinescens*	*Exocarpos aphyllus*	*Proteaceae* sp. 1	*Grass* sp. 1	*Brassica tournefortii*	*Nitraria billardierei*
*Scaevola spinescens*	*Goodenia pusilliflora*	*Rhagodia eremaea*	*Grass* sp. 2	*Bromus arenarius*	*Solanum orbiculatum*
*Sisymbrium erysimoides*	*Grass* sp. 2	*Santalum lanceolatum*	*Grevillea* sp. 1	*Scaevola tomentosa*	
*Sisymbrium sp*.	*Hyalosperma semisterile*		*Pittosporum angustifolium*	*Sida sp*.	
*Spergularia sp*.	*Moraea setifolia*		*Templetonia egena*	*Tribulus terrestris*	
*Stenopetalum sp*.	*Pauridia glabella var. glabella*				
*Mallee* sp. 2	*Senna artemisioides filifolia*				
*Herb* sp. 1	*Senna sp*.				
*Mallee* sp. 1	*Silene apetala*				
*Grass* sp. 1	Sp. 1				
	Sp. 2				
	*Velleia arguta*				
	*Vittadinia megacephala*				

**TABLE A8:** The mean NDVI values on boodie warrens at three sites, Yookamurra, Matuwa, and Faure

	Warren	Control
Yookamurra		
Warren diameter	−0.03 ± 0.02	−0.04 ± 0.02
Warren diameter + 10 m buffer zone	−0.03 ± 0.01	−0.04 ± 0.01
Matuwa		
Warren diameter	−0.12 ± 0.02	−0.13 ± 0.02
Warren diameter + 10 m buffer zone	−0.12 ± 0.01	−0.12 ± 0.01
Faure		
Warren diameter	−0.04 ± 0.02	−0.03 ± 0.02
Warren diameter + 5 m buffer zone	−0.03 ± 0.02	−0.03 ± 0.02

## References

[ece37218-bib-0001] Abbott, I. (2008). Historical perspectives of the ecology of some conspicuous vertebrate species in south‐west Western Australia. Conservation Science Western Australia, 6, 1–214.

[ece37218-bib-0002] Andersen, D. C. , & Macmahon, J. A. (1985). Plant succession following the Mount St‐Helens volcanic eruption: Facilitation by a burrowing rodent, *Thomomys talpoides* . American Midland Naturalist, 114, 62–69.

[ece37218-bib-0003] Ashton, L. J. , & McKenzie, N. J. (2001). Conversion of the Atlas of Australian Soils to the Australian soil classification, Canberra, Australia: CSIRO Land and Water.

[ece37218-bib-0004] Bates, D. , Maechler, M. , Bolker, B. , & Walker, S. (2015). Fitting linear mixed‐effects models using lme4. Journal of Statistical Software, 67, 1–48.

[ece37218-bib-0005] Beals, S. C. , Hartley, L. M. , Prevéy, J. S. , & Seastedt, T. R. (2014). The effects of black‐tailed prairie dogs on plant communities within a complex urban landscape: An ecological surprise? Ecology, 95, 1349–1359. 10.1890/13-0984.1 25000766

[ece37218-bib-0006] Bryce, R. , van der Wal, R. , Mitchell, R. , & Lambin, X. (2013). Metapopulation dynamics of a burrowing herbivore drive spatio‐temporal dynamics of riparian plant communities. Ecosystems, 16, 1165–1177.

[ece37218-bib-0007] Burbidge, A. A. , Harrison, P. L. , & Woinarski, J. (2014). The action plan for Australian mammals 2012, Melbourne, Australia: CSIRO Publishing.

[ece37218-bib-0008] Burbidge, A. A. , McKenzie, N. L. , Brennan, K. E. C. , Woinarski, J. C. Z. , Dickman, C. R. , Baynes, A. , Gordon, G. , Menkhorst, P. W. , & Robinson, A. C. (2008). Conservation status and biogeography of Australia's terrestrial mammals. Australian Journal of Zoology, 56, 411–422.

[ece37218-bib-0009] Burbidge, A. A. , Short, J. , & Fuller, P. J. (2007). Relict *Bettongia lesueur* warrens in Western Australian deserts. Australian Zoologist, 34, 97–103.

[ece37218-bib-0010] Bureau of Meteorology (2019). Climate statistics for Australian locations [Online]. Commonwealth of Australia. Retrieved from http://www.bom.gov.au/climate/data/

[ece37218-bib-0011] Butler, D. R. (1995). Zoogeomorphology: Animals as geomorphic agents, New York, United States of America: Cambridge University Press.

[ece37218-bib-0012] Catano, C. P. , & Stout, I. J. (2015). Functional relationships reveal keystone effects of the gopher tortoise on vertebrate diversity in a longleaf pine savanna. Biodiversity and Conservation, 24, 1957–1974.

[ece37218-bib-0013] Chapman, T. F. (2015). Comparison of soils and plants on the active and relic parts of a recolonised burrowing bettong (*Bettongia lesueur*) warren. Pacific Conservation Biology, 21, 298–306.

[ece37218-bib-0014] CSBP Lab (2019). CSBP lab methods. CSBP Lab.

[ece37218-bib-0015] CSIRO and Bureau of Meteorology (2015). Climate change in Australia information for Australia's natural resource management regions: Technical report. Australia. CSIRO and Bureau of Meteorology.

[ece37218-bib-0016] Davidson, A. D. , Detling, J. K. , & Brown, J. H. (2012). Ecological roles and conservation challenges of social, burrowing, herbivorous mammals in the world's grasslands. Frontiers in Ecology and the Environment, 10, 477–486.

[ece37218-bib-0017] Davidson, A. D. , & Lightfoot, D. C. (2006). Keystone rodent interactions: Prairie dogs and kangaroo rats structure the biotic composition of a desertified grassland. Ecography, 29, 755–765. 10.1111/j.2006.0906-7590.04699.x

[ece37218-bib-0018] Davidson, A. D. , & Lightfoot, D. C. (2008). Burrowing rodents increase landscape heterogeneity in a desert grassland. Journal of Arid Environments, 72, 1133–1145.

[ece37218-bib-0019] Dawson, S. J. , Broussard, L. , Adams, P. J. , Moseby, K. E. , Waddington, K. I. , Kobryn, H. T. , Bateman, P. W. , & Fleming, P. A. (2019). An outback oasis: The ecological importance of bilby burrows. Journal of Zoology, 308, 149–163.

[ece37218-bib-0020] de Caceres, M. , & Legendre, P. (2009). Associations between species and groups of sites: Indices and statistical inference. Ecology, 90, 3566–3574. 10.1890/08-1823.1 20120823

[ece37218-bib-0021] Ellis, E. C. , Goldewijk, K. K. , Siebert, S. , Lightman, D. , & Ramankutty, N. (2010). Anthropogenic transformation of the biomes, 1700 to 2000. Global Ecology and Biogeography, 19, 589–606.

[ece37218-bib-0022] Facelli, J. M. , & Chesson, P. (2008). Cyclic dormancy, temperature and water availability control germination of *Carrichtera annua*, an invasive species in chenopod shrublands. Austral Ecology, 33, 324–328.

[ece37218-bib-0023] Finlayson, G. R. , & Moseby, K. M. (2004). Managing confined populations: The influence of density on the home range and habitat use of reintroduced burrowing bettongs (*Bettongia lesueur*). Wildlife Research, 31, 457–463. 10.1071/WR03035

[ece37218-bib-0024] Fleming, P. A. , Anderson, H. , Prendergast, A. S. , Bretz, M. R. , Valentine, L. E. , & Hardy, G. E. S. (2014). Is the loss of Australian digging mammals contributing to a deterioration in ecosystem function? Mammal Review, 44, 94–108.

[ece37218-bib-0025] Grant, W. E. , French, N. R. , & Folse, L. J. (1980). Effects of pocket gopher mounds on plant production in shortgrass prairie ecosystems. The Southwestern Naturalist, 25, 215–224.

[ece37218-bib-0026] Hagenah, N. , Bennett, N. C. , & Kitchener, A. (2013). Mole rats act as ecosystem engineers within a biodiversity hotspot, the Cape Fynbos. Journal of Zoology, 289, 19–26.

[ece37218-bib-0027] Haussmann, N. S. , Louw, M. A. , Lewis, S. , Nicol, K. J. H. , van der Merwe, S. , & le Roux, P. C. (2018). Ecosystem engineering through aardvark (*Orycteropus afer*) burrowing: Mechanisms and effects. Ecological Engineering, 118, 66–72.

[ece37218-bib-0028] IUCN, SSC (2013). Guidelines for reintroductions and other conservation translocations, Gland, Switzerland: IUCN Species Survival Commission.

[ece37218-bib-0029] Jones, C. G. , Lawton, J. H. , & Shachak, M. (1994). Organisms as ecosystem engineers. Oikos, 69, 373–386.

[ece37218-bib-0030] Kerley, G. I. H. , Whitford, W. G. , & Kay, F. R. (2004). Effects of pocket gophers on desert soils and vegetation. Journal of Arid Environments, 58, 155–166.

[ece37218-bib-0031] Komonen, M. , Komonen, A. , & Otgonsuren, A. (2003). Daurian pikas (*Ochotona daurica*) and grassland condition in eastern Mongolia. Journal of Zoology, 259, 281–288.

[ece37218-bib-0032] Kurek, P. , Kapusta, P. , & Holeksa, J. (2013). Burrowing by badgers (*Meles meles*) and foxes (*Vulpes vulpes*) changes soil conditions and vegetation in a European temperate forest. Ecological Research, 29, 1–11.

[ece37218-bib-0033] Larson, D. L. (2003). Native weeds and exotic plants: Relationships to disturbance in mixed‐grass prairie. Plant Ecology, 169, 317–333.

[ece37218-bib-0034] Leutner, B. , Horning, N. , & Schwakb‐Willmann, J. (2019). RStoolbox: Tools for remote sensing data analysis. R package version 0.2.6. https://CRAN.R‐project.org/package=RStoolbox.

[ece37218-bib-0035] Liebman, M. , Mohler, C. L. , & Staver, C. P. (2001). Ecological management of agricultural weeds, Cambridge, United Kingdom: Cambridge University Press.

[ece37218-bib-0036] Mallen‐Cooper, M. , Nakagawa, S. , & Eldridge, D. J. (2019). Global meta‐analysis of soil‐disturbing vertebrates reveals strong effects on ecosystem patterns and processes. Global Ecology and Biogeography, 28, 661–679.

[ece37218-bib-0037] Moorhead, D. L. , Fisher, F. M. , & Whitford, W. G. (1988). Cover of spring annuals on nitrogen‐rich kangaroo rat mounds in a Chihuahuan Desert grassland. The American Midland Naturalist, 120, 443–447.

[ece37218-bib-0038] Morton, S. R. , Stafford Smith, D. M. , Dickman, C. R. , Dunkerley, D. L. , Friedel, M. H. , McAllister, R. R. J. , Reid, J. R. W. , Roshier, D. A. , Smith, M. A. , Walsh, F. J. , Wardle, G. M. , Watson, I. W. , & Westoby, M. (2011). A fresh framework for the ecology of arid Australia. Journal of Arid Environments, 75, 313–329.

[ece37218-bib-0039] Munro, N. T. , McIntyre, S. , Macdonald, B. , Cunningham, S. A. , Gordon, I. J. , Cunningham, R. B. , & Manning, A. D. (2019). Returning a lost process by reintroducing a locally extinct digging marsupial. PeerJ, 7, e6622. 10.7717/peerj.6622 31179166 PMC6542348

[ece37218-bib-0040] Newell, J. (2008). The role of the reintroduction of Greater Bilbies (*Macrotis lagotis*) and Burrowing Bettongs (*Bettongia lesueur*) in the ecological restoration of an arid ecosystem: Foraging diggings, diet, and soil seed banks. Doctor of Philosophy, University of Adelaide.

[ece37218-bib-0041] Noble, J. C. , Müller, W. J. , Detling, J. K. , & Pfitzner, G. H. (2007). Landscape ecology of the burrowing bettong: Warren distribution and patch dynamics in semiarid eastern Australia. Austral Ecology, 32, 326–337.

[ece37218-bib-0042] Oksanen, J. , Blanchet, F. G. , Friendly, M. , Kindt, R. , Legendre, P. , McGlinn, D. , Minchin, P. R. , O'Hara, R. B. , Simpson, G. L. , Solymos, P. , Stevens, M. H. H. , Szoecs, E. , & Wagner, H. (2019). vegan: Community ecology package. 2.5‐6 ed. https://CRAN.R‐project.org/package=vegan

[ece37218-bib-0043] Palmer, B. J. , Valentine, L. E. , Page, M. , & Hobbs, R. J. (2020). Translocations of digging mammals and their potential for ecosystem restoration: A review of goals and monitoring programmes. Mammal Review, 50, 382–398. 10.1111/mam.12208

[ece37218-bib-0044] Parer, I. , & Libke, J. A. (1985). Distribution of rabbit, *Oryctolagus cuniculus*, warrens in relation to soil type. Australian Wildlife Research, 12, 387–405.

[ece37218-bib-0045] Parsons, M. A. , Barkley, T. C. , Rachlow, J. L. , Johnson‐Maynard, J. L. , Johnson, T. R. , Milling, C. R. , Hammel, J. E. , & Leslie, I. (2016). Cumulative effects of an herbivorous ecosystem engineer in a heterogeneous landscape. Ecosphere, 7, e01334‐n/a. 10.1002/ecs2.1334

[ece37218-bib-0046] Pike, D. A. , & Mitchell, J. C. (2013). Burrow‐dwelling ecosystem engineers provide thermal refugia throughout the landscape. Animal Conservation, 16, 694–703.

[ece37218-bib-0047] R Core Team (2019). R: A language and environment for statistical computing. R Foundation for Statistical Computing. https://www.R‐project.org/.

[ece37218-bib-0048] Read, J. L. , Carter, J. , Moseby, K. M. , & Greenville, A. (2008). Ecological roles of rabbit, bettong and bilby warrens in arid Australia. Journal of Arid Environments, 72, 2124–2130.

[ece37218-bib-0049] Ride, W. D. L. , Mess, G. F. , Douglas, A. M. , Royce, R. D. , & Tyndale‐Biscoe, C. H. (1962). The results of an expedition to Bernier and Dorre Islands Shark Bay, Western Australia in July, 1959, Western Australia: Fisheries Department.

[ece37218-bib-0050] Sander, U. , Short, J. , & Turner, B. (1997). Social organisation and warren use of the burrowing bettong, *Bettongia lesueur* (Macropodoidea: Potoroidae). Wildlife Research, 24, 143–157.

[ece37218-bib-0051] Seddon, P. J. (2010). From reintroduction to assisted colonization: Moving along the conservation translocation spectrum. Restoration Ecology, 18, 796–802.

[ece37218-bib-0052] Seddon, P. J. , Griffiths, C. J. , Soorae, P. S. , & Armstrong, D. P. (2014). Reversing defaunation: Restoring species in a changing world. Science, 345, 406. 25061203 10.1126/science.1251818

[ece37218-bib-0053] Sherrod, S. K. , & Seastedt, T. R. (2001). Effects of the northern pocket gopher (*Thomomys talpoides*) on alpine soil characteristics, Niwot Ridge, CO. Biogeochemistry, 55, 195–218.

[ece37218-bib-0054] Short, J. , & Turner, B. (1999). Ecology of burrowing bettongs, *Bettongia lesueur* (Marsupialia: Potoroidae), on Dorre and Bernier Islands, Western Australia. Wildlife Research, 26, 651–669. 10.1071/WR98039

[ece37218-bib-0055] Smith, D. M. S. , & Morton, S. R. (1990). A framework for the ecology of arid Australia. Journal of Arid Environments, 18, 255–278.

[ece37218-bib-0056] Stodart, E. (1966). Observations on the behaviour of the marsupial *Bettongia lesueuri* (Quoy & Gaimard) in an enclosure. Wildlife Research, 11, 91–99.

[ece37218-bib-0057] Torres‐Díaz, C. , Gómez‐González, S. , Torres‐Morales, P. , & Gianoli, E. (2011). Soil disturbance by a native rodent drives microhabitat expansion of an alien plant. Biological Invasions, 14, 1211–1220.

[ece37218-bib-0058] Valkó, O. , Tölgyesi, C. , Kelemen, A. , Bátori, Z. , Gallé, R. , Rádai, Z. , Bragina, T. M. , Bragin, Y. A. , Deák, B. (2021). Steppe Marmot (Marmota bobak) as ecosystem engineer in arid steppes. Journal of Arid Environments, 184, 104244. 10.1016/j.jaridenv.2020.104244.

[ece37218-bib-0059] van der Waal, C. , de Kroon, H. , van Langevelde, F. , de Boer, W. F. , Heitkonig, I. M. , Slotow, R. , Pretorius, Y. , & Prins, H. H. (2016). Scale‐dependent bi‐trophic interactions in a semi‐arid savanna: How herbivores eliminate benefits of nutrient patchiness to plants. Oecologia, 181, 1173–1185. 10.1007/s00442-016-3627-0 27094543 PMC4954840

[ece37218-bib-0060] Verdon, S. J. , Gibb, H. , & Leonard, S. W. J. (2016). Net effects of soil disturbance and herbivory on vegetation by a re‐established digging mammal assemblage in arid zone Australia. Journal of Arid Environments, 133, 29–36.

[ece37218-bib-0061] Walker, F. M. , Taylor, A. C. , & Sunnucks, P. (2007). Does soil type drive social organization in southern hairy‐nosed wombats? Molecular Ecology, 16, 199–208. 17181731 10.1111/j.1365-294X.2006.03131.x

[ece37218-bib-0062] Warton, D. I. , & Hui, F. K. C. (2011). The arcsine is asinine: The analysis of proportions in ecology. Ecology, 92, 3–10. 21560670 10.1890/10-0340.1

[ece37218-bib-0063] Wesche, K. , Nadrowski, K. , & Retzer, V. (2007). Habitat engineering under dry conditions: The impact of pikas (*Ochotona pallasi*) on vegetation and site conditions in southern Mongolian steppes. Journal of Vegetation Science, 18, 665–674.

[ece37218-bib-0064] Wheeler, H. C. , & Hik, D. S. (2013). Arctic ground squirrels *Urocitellus parryiias* drivers and indicators of change in northern ecosystems. Mammal Review, 43, 238–255.

[ece37218-bib-0065] Whittington‐Jones, G. M. , Bernard, R. T. F. , & Parker, D. M. (2011). Aardvark burrows: A potential resource for animals in arid and semi‐arid environments. African Zoology, 46, 362–370.

